# Fabrication of Radially Symmetric Graded Porous Silicon using a Novel Cell Design

**DOI:** 10.1038/srep24864

**Published:** 2016-04-22

**Authors:** Mingrui Zhao, Manish Keswani

**Affiliations:** 1 Chemical and Environmental Engineering, University of Arizona, Tucson, AZ, USA; 2 Materials Science and Engineering, University of Arizona, Tucson, AZ, USA

## Abstract

A contactless method using a novel design of the experimental cell for formation of porous silicon with morphological gradient is reported. Fabricated porous silicon layers show a large distribution in porosity, pore size and depth along the radius of the samples. Symmetrical arrangements of morphology gradient were successfully formulated radially on porous films and the formation was attributed to decreasing current density radially inward on the silicon surface exposed to Triton^®^ X-100 containing HF based etchant solution. Increasing the surfactant concentration increases the pore depth gradient but has a reverse effect on the pore size distribution. Interestingly, when dimethyl sulfoxide was used instead of Triton^®^ X-100 in the etchant solution, no such morphological gradients were observed and a homogeneous porous film was formed.

The porosification of silicon is conventionally obtained by anodic dissolution of a silicon wafer in a fluoride-based electrolyte solution, as first described by Uhlir in 1958^1^. Porous silicon (PSi) materials are highly biocompatible and biodegradable^2^, exhibiting versatile electronic properties and have generated increasing interests for a wide spectrum of potential applications in semiconductor technology^3^,^4^, chemical and biological sensors^5^,^6^,^7^,^8^, optoelectronic materials and other fields of sciences^9^,^10^,^11^,^12^,^13^,^14^. PSi is also appealing due to high tunability of its properties via manipulation of processing conditions^15^. Parameters like dopant type and concentration, etching time, applied current density and voltage, electrolyte composition (HF concentration, addition of surfactant or organic solvent) and light intensity are all considered to play a role in modifying porosification, pore size distribution and surface morphology^16^.

Generally PSi with uniform size distribution of pores can be fabricated by using various methods, employing new designs of the experimental set-ups and using chemical formulations with different additives^15^. More recently, PSi with a gradient in porosity, pore size and depth was developed with the purpose of fulfilling special needs in applications related to biomaterials, biosensors and bioadhesion research, where gradient/graded surfaces are considered as valuable tools^17^,^18^. As an example, by utilizing gradient/graded PSi, characterization of cell-surface interactions as systematic screening and optimization of surface parameters can be processed in a single sample, which will reduce the sample numbers and variation between experiments^19^.

Methods illustrating fabrication of PSi films with pore size gradient in an asymmetrical arrangement have been discussed recently^20^,^21^,^22^. In the setup demonstrated by Collins *et al*. and Khung *et al*., a Pt electrode was placed perpendicular to the silicon surface at one end of the sample, thus causing the potential at silicon-electrolyte interface to be a function of the distance between working and counter electrodes, resulting in a current density gradient on the sample surface from one end to the other^20^,^21^. In another study, Karlsson *et al*. proposed a back-side etching method by using two silicon wafers as electrodes. PSi with varying gradient of morphology was fabricated due to inhomogeneous current density on the back side of the anode, where the current density was lower further away from the edges of the wafers^22^.

Herein, we are proposing a new design that is a modification of the geometry we have reported before for fabrication of PSi with morphological gradient^23^. This innovative and scalable method for PSi formation relies on redox reactions occurring at silicon-solution interfaces. The most commonly used method to prepare PSi is by anodic (electrochemical) etching in HF-based solutions^24^. Alternatively, it can be prepared without any electrochemical bias by stain etching, where oxidative dissolution takes place in the presence of strong oxidizing agent like HNO_3_^25^,^26^. In comparison, our technique does not require the silicon wafer to be an electrode and PSi is formed under no external anodic bias (to silicon) and without employing oxidizing agents in the etchant solution. Modifications of the previous experimental setup and use of suitable additives in the HF etchant solution allowed the formation of PSi with a wide distribution of porosity, pore size and pore depth and in a radially symmetrical arrangement, which can be expected to meet the increasing demands for special applications in biosensors, filters and optoelectronic materials. Since, in our process, the only contact of the silicon is with the etchant and deposition solutions (NiSO_4_/H_3_BO_3_), the process is completely scalable.

## Results

**Figure 1:**
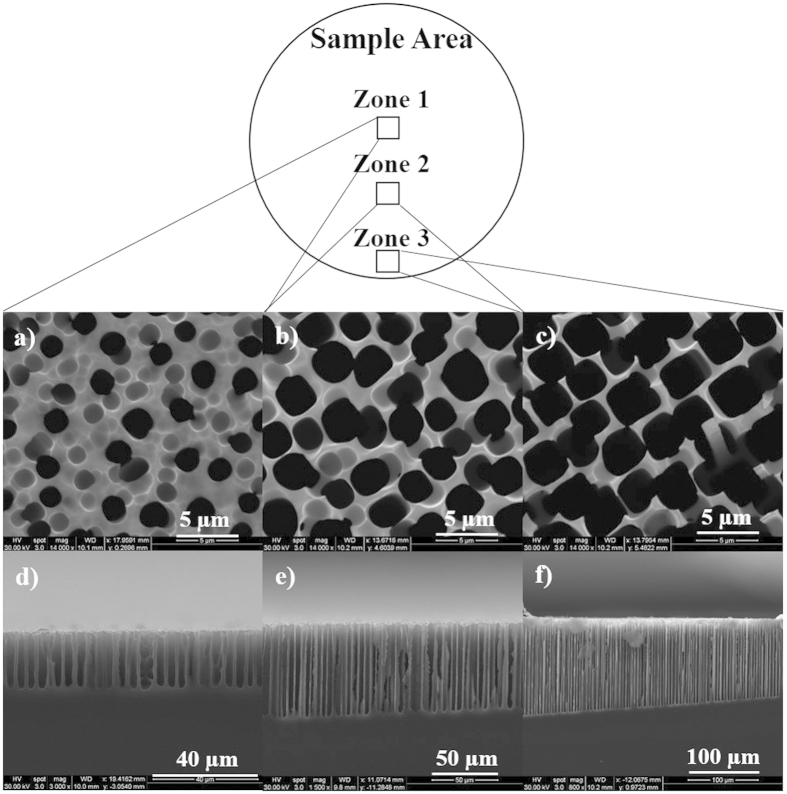
Surface morphology of porous silicon structures formed using etchant solution containing 1E-1% Triton^®^ X-100. SEM images showing (**a**–**c**) top view and (**d–f**) cross sectional view of porous films at Zone 1, 2 and 3.

**Figure 2:**
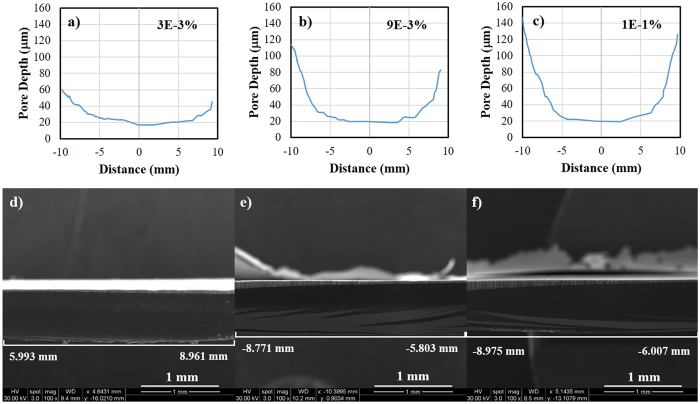
Comparison of symmetric morphological gradient for porous films obtained using different conditions. Plots and SEM images showing pore depth as a function of distance along the diameter of the circular region of the samples prepared using different concentration of Triton^®^ X-100 in etchant solution. (**a**) and (**d**) 3E-3%; (**b**) and (**e**) 9E-3%; (**c**) and (**f**) 1E-1%. Center of the sample was set as zero point.

To characterize the morphology of porous films, samples were inspected using scanning electron microscopy (SEM). Figure 1a through f show the morphological gradient feature of fabricated PSi using 1E-1 weight% (value above CMC) Triton^®^ X-100 in aqueous HF solution. SEM images for the top and cross sectional view were taken at three different locations (marked as Zone 1, 2 and 3) along the radius of the circular region. A statistical analysis of the micrographs was performed using ImageJ^®^ software to calculate the average pore diameter (*d*_*avg*_), porosity (*p*) and average pore depth (*h*_*avg*_) (see Supplementary Fig. 1). Comparing the surface properties of films at Zone 1 and 2, the values of *d*_*avg*_, *h*_*avg*_, and *p* increased from 1.8 ± 0.2 μm, 23 ± 1 μm and 19.3% to 3.1 ± 0.4 μm, 58 ± 5 μm and 52.4%, respectively (see Supplementary Table 1). When moving further to the edge of the sample at Zone 3, larger values of *d*_*avg*_, *h*_*avg*_, and *p* were observed and calculated to be 3.4 ± 0.5 μm, 127 ± 7 μm and 63.2% respectively. Clearly, porosity, pore size and depth of films dramatically increased when moving from Zone 1 to 3 due to increase of current density. In order to understand and establish relationship between processing parameters and surface properties of porous films, similar experiments were conducted by adding varying amounts of Triton^®^ X-100 to investigate the effect of surfactant concentration on pore formation using this method. SEM micrographs of samples prepared using aqueous HF solutions containing 3E-3weight% and 9E-3weight% (values below CMC) Triton^®^ X-100 show analogous morphological characteristics with increasing pore size and depth and porosity of films from the center to the edge (see Supplementary Figs S2 and S3). Calculated values of *d*_*avg*_, *h*_*avg*_, and *p* for porous films of these samples are provided in Table S1 (see Supplementary Table 1). Figure 2a through c show pore depth as a function of distance along the diameter (from one end to the other) of the circular region of the sample prepared using different concentrations of Triton^®^ X-100 in the etchant solution, where the center was set as zero point. The plots show some symmetry along the center with continuous pore depth gradients (increasing depths from center to the edge) on both sides. SEM images captured at lower magnification and illustrating the gradients for a smaller section of the sample are shown in Fig. 2d through f. These results further indicate that lowering Triton^®^ X-100 concentration from 1E-1 to 3E-3 weight% significantly reduces the maximum pore depth that can be achieved and lowers the gradient. It may be noted that the pore depth at the center was unaffected by Triton^®^ X-100 concentration.

**Figure 3:**
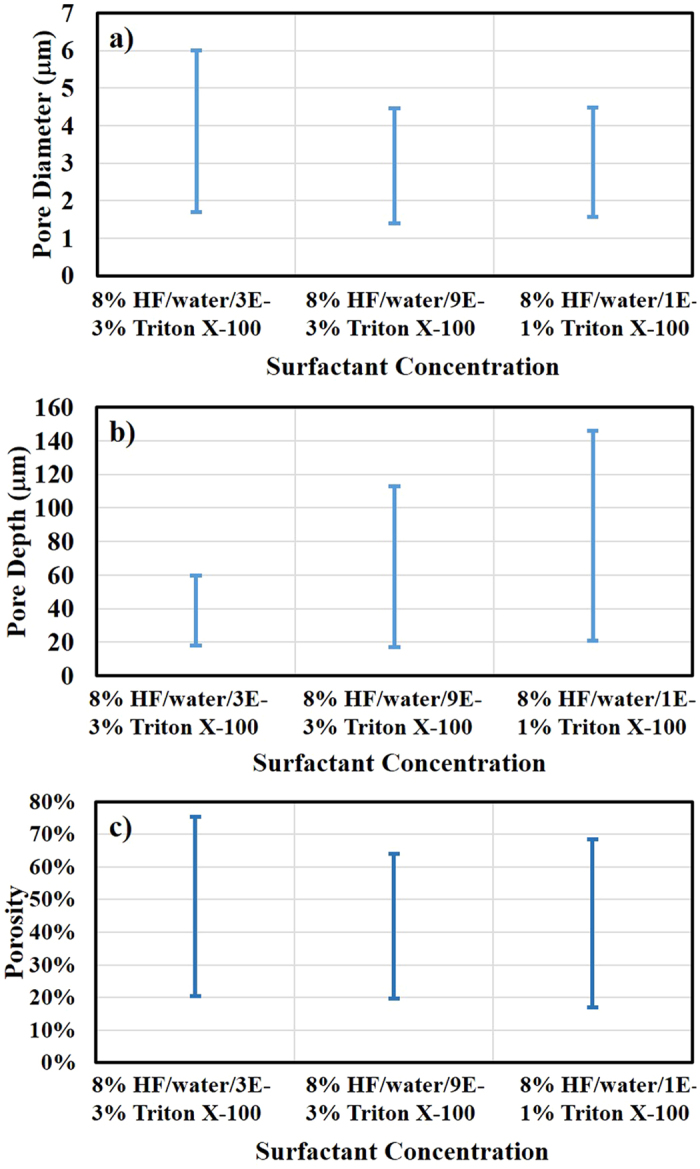
Comparison of surface parameters for porous films obtained using different conditions. Plots for (**a**) pore diameter, (**b**) pore depth and (**c**) porosity distribution of porous films prepared using different concentrations of Triton^®^ X-100 in HF based etchant solutions.

Effect of Triton^®^ X-100 concentration on pore diameter, pore depth and porosity distribution of gradient PSi samples was examined and plotted in Fig. 3a–c. The largest distribution of pore diameter ranged from 1.8 to 6 μm and was obtained using 3E-3 weight% Triton^®^ X-100 in the etchant solution. Increase in Triton^®^ X-100 concentration to 9E-3 weight% shrunk the range of pore diameter distribution but further increasing in concentration did not have any significant effect. By contrast, the distribution of pore depth followed the reverse trend. Samples prepared using 1E-1 weight% Triton^®^ X-100 exhibited the sharpest gradient with the pore depth ranging from 20–142 μm and the gradient reduced with decrease in Triton^®^ X-100 concentration. There was no clear effect of surfactant concentration on porosity as the distribution was similar and roughly in the range of 20–70% for all three concentration of Triton^®^ X-100.

Adsorption of surfactant onto silicon-liquid interface is expected to modify the Helmholtz layer and space charge layer^27^. At this interface, surfactant molecules tend to attach their hydrophobic tails to hydrogen-terminated silicon surface and stick the polar heads out with water, thereby affecting both the silicon dissolution kinetics and local electrostatic properties and electrochemical behavior^28^. Higher concentration of surfactant leads to lower surface tension, which improves the wettability and allows penetration of HF based etchant solution into the bottom of porous films to form deeper pores. Since, a constant current density was applied for the same period of time in all cases, equal amount of silicon should be removed from each sample. In our experiments, wettability of solutions containing smaller amount of surfactant might not be high enough which may cause penetration issues, leading to smaller pore depths but larger diameters. For higher surfactant concentrations, increase in wettability allows formation of deeper pores but causes a decrease in pore size thereby complying with equal amount of silicon etching from each sample.

Studies were also carried out using 84% dimethyl sulfoxide (DMSO)/8%H_2_O/8%HF (all by weight) solution as the etchant solution and keeping all other experimental conditions the same. Interestingly, morphological gradient was not observed for porous films formed in this case. A homogenous porous layer was formed in the circular area instead, as observed by visual inspection of the samples and confirmed using SEM analysis. In Fig. S4 (see Supplementary Fig. 4), a color gradient along the radius of the sample fabricated using aqueous Triton^®^ X-100 solution can be noticed (Fig. S4a), while samples prepared using DMSO/H_2_O/HF solutions show consistent color everywhere on the sample (Supplementary Fig. 4b,c). SEM images of DMSO/H_2_O/HF samples further illustrate the homogeneous formation of porous film, suggesting that the expected current density gradient was not developed on the reacting surface (see Supplementary Fig. 4d–i). A more comprehensive comparison of porous film characteristics under different etchant solution conditions have been plotted and shown in Fig. S5 (see Supplementary Fig. 5), which further confirms our observation. The reason behind formation of the homogenous porous layer using DMSO based etchant solutions is unknown at this stage, although it seems that DMSO and Triton^®^ X-100 may be playing a different role beyond just improving the wettability of the silicon surface. More detailed studies to understand this behaviour will be carried out in the near future.

In the current work, we have successfully developed a novel cell design for fabrication of graded PSi using a contactless process employing HF based etchant solution containing Triton^®^ X-100. The design allows decreasing current density radially inward along the sample surface, which results in morphological variation in the structure of the porous films. Surface properties including porosity, pore size and pore depth can be modified via manipulation of the surfactant concentration. The graded porous films showed large gradients of surface morphologies in radial and symmetrical arrangements, offering promise for practical applications in sensors, biomaterials, and photocatalysis.

## Methods

**Figure 4:**
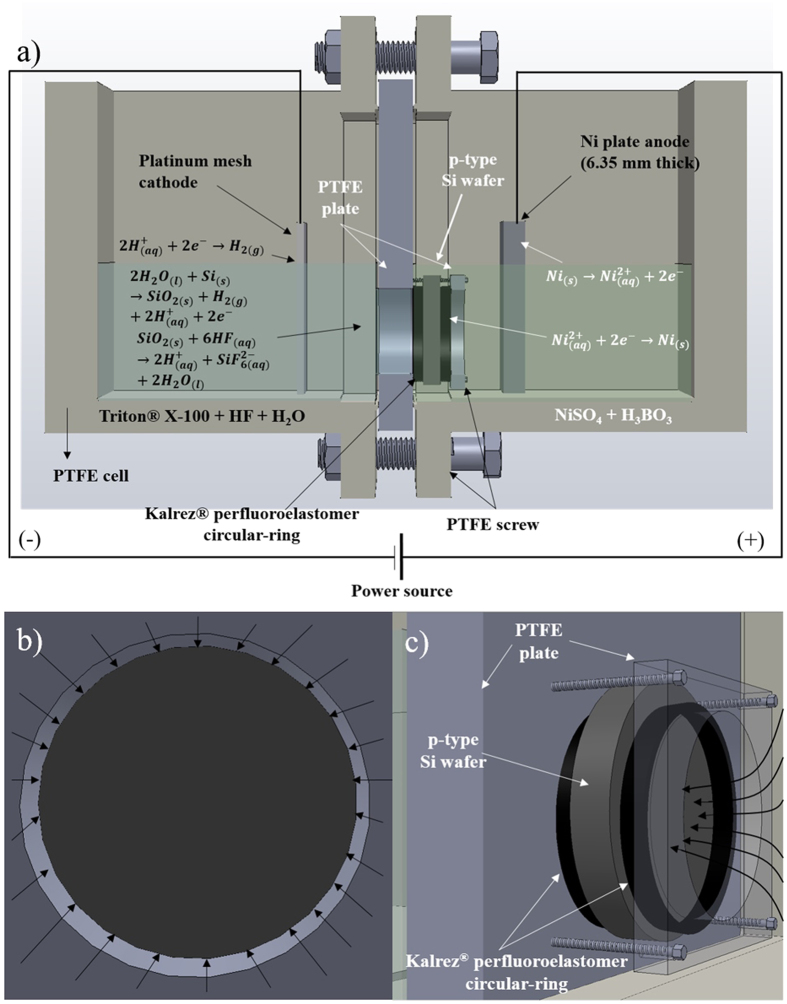
Schematic and working mechanism of the new cell design. (**a**) Schematic of modified dual compartment PTFE cell used for generating graded porous silicon. Silicon wafer placed and sealed between the two Teflon^®^ plates. The anodic and cathodic reactions of silicon dissolution and nickel deposition occur continuously and concurrently at the silicon-solution interfaces. (**b**,**c**) Flow of current in the wafer results in a current density gradient radially along the reacting surface.

A schematic illustrating the proposed process is shown in Fig. 4a. The experimental details are provided in the Electronic Supplementary Information. A plate with a circular opening is placed and sealed in a cell consisting of two chambers, with all parts and accessories contacting the sample and the solutions made of polytetrafluoroethylene (PTFE, Teflon^®^). The target silicon sample of 14 Ω-cm resistivity is placed onto the plate and sealed with another smaller plate such that only a circular region of silicon is exposed to the HF (8 weight%) based etchant solution while the rest of the sample surface is in contact with the aqueous deposition solution containing 1 M NiSO_4_ and 0.5 M H_3_BO_3_. Two electrodes, Ni plate anode and Pt mesh cathode, are immersed in the deposition and etchant solutions, respectively, parallel and facing the wafer surface with a constant current density of 10 mA/cm^2^ applied between them for 2 h. Such a configuration allows a decreasing current density radially inward in the circular region exposed to the etchant solution. Further, the decrease in current density can be expected to be constant due to uniform contact with the solutions. A non-ionic surfactant, Triton^®^ X-100 was added at different concentrations (above and below critical micelle concentration (CMC = 12E-3–15E-3 weight% at 25 °C)) to the etchant solution to develop the porous films. In this process, silicon in contact with etchant solution undergoes a two-step dissolution, the first reaction is electrochemical oxidation from Si to SiO_2_ while the second one is the chemical etching of SiO_2_^23^. The electrons generated from the first reaction are conducted through the bulk silicon wafer to be consumed by Ni^2 + ^, which then gets deposited uniformly in the form of a thin nickel film on the entire exposed surface. The flow of current from different directions, illustrated in Fig. 4b,c allows a current density gradient to be developed radially along the surface, thereby creating a symmetrical distribution of graded porous films along the radius in the circular area exposed to HF solution.

## Additional Information

**How to cite this article**: Zhao, M. and Keswani, M. Fabrication of Radially Symmetric Graded Porous Silicon using a Novel Cell Design. *Sci. Rep.* **6**, 24864; doi: 10.1038/srep24864 (2016).

## Supplementary Material

Supplementary Information
