# Supplementary material for: Fabrication of Radially Symmetric Graded Porous Silicon using a Novel Cell Design
Source: Sci Rep. 2016 Apr 22;6:24864. doi: 10.1038/srep24864 (PMC4840451; doi:10.1038/srep24864)
Supplement: Supplementary Information [file srep24864-s1.pdf]

Supplementary Information

Fabrication of Radially Symmetric Graded Porous Silicon using a Novel Cell Design

Mingrui Zhao [a] and Manish Keswani [b, *]

[a] Chemical and Environmental Engineering, University of Arizona, Tucson, AZ, USA
[b] Materials Science and Engineering, University of Arizona, Tucson, AZ, USA
[*] Corresponding Author. Address: 1235 E. James E. Rogers Way, Tucson, AZ 85721, USA; E-mail: manishk@email.arizona.edu; Fax: +1-520-621-8059

Materials and Methods

De-ionized (DI) water of 18 MΩ-cm resistivity was used in the preparation of solutions and for rinsing of the samples. VLSI grade isopropyl alcohol (99%) and hydrofluoric acid (49%) were obtained from Honeywell Inc. Dimethyl sulfoxide (DMSO, >99%) and boric acid (>99%) were purchased from Fisher Scientific Inc. Nickel sulfate hexahydrate (99%) and 4-(1,1,3,3-Tetramethylbutyl)phenyl-polyethylene glycol (Triton® X-100, labortary grade, 98%) were procured from Sigma Aldrich. The critical micelle concentration of this non-ionic surfactant is 0.2 to 0.9 mM, corresponding to 12E-3 to 15E-3% (by weight) at 20 – 25 °C. The samples used for porous silicon formation were p-type Si (100) wafers with different resistivity (8 and 14 Ω cm). Nickel plate (99.5%) with dimensions of 50 mm x 50 mm x 6.35 mm was used as the anode, while platinum mesh (99.9%) of size 50 x 50 mm was used as the cathode. A power source from B&K Precision Corporation (current and potential limit of 5 A and 41 V) was used to apply current to the system.

Fabrication of porous silicon

The experimental setup includes a two chamber polytetrafluoroethylene (PTFE, Teflon) cell, target silicon wafer and two Teflon plates of different sizes. The larger Teflon plate with a circular opening is placed and fixed between the two chambers. The silicon wafer is placed and sealed between the two Teflon plates using two Kalrez® perfluoroelastomer circular-rings. Before each experiment, silicon wafer was pretreated with piranha (1H_2O_2 (30%): 4H_2SO_4 (96%) by volume, 5 min), diluted HF (1HF (49%): 50H_2O, 1 min), ammonia-peroxide mixture (1NH4OH (29%): 1H_2O_2 (30%) :5H_2O, 5 min) and diluted HF (1HF (49%): 50H_2O, 1 min). The wafer was then coated with Ni seed layer using the method previously described (ref. 23). The Ni coated side contacted the deposition solution, which contained 1.0 M nickel sulfate and 0.5 M boric acid. The etchant solution consisted of 8% HF and 92% H_2O (by weight) with varying concentrations of Triton® X-100 or 8% HF, 8% water and 84% DMSO (by weight). Ni anode and Pt mesh cathode

were immersed in deposition solution and etchant solution, respectively. A constant current density of 10 mA/cm^2 was applied between the cathode and anode for a period of 2 h. High resolution scanning electron microscopy (SEM, FEI Inspec-S50) images of the porous films were captured and analyzed using ImageJ® software for characterization of pore dimensions and surface morphology.

Surface parameter analysis

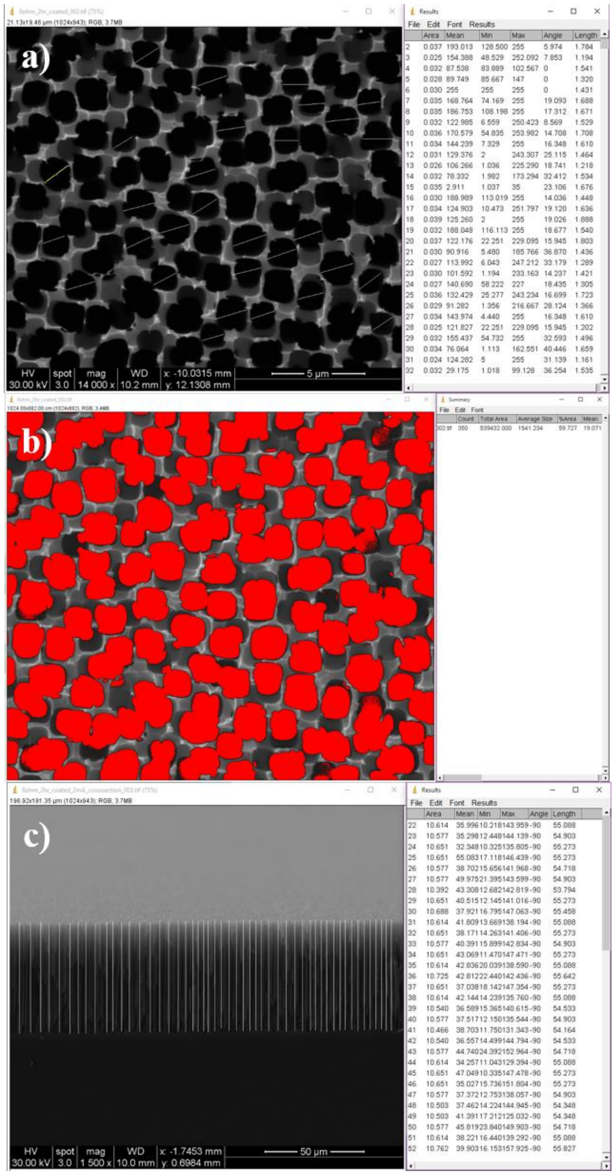

Supplementary Figure 1. Analysis of pore deminsions using ImageJ® software. Characterization and calculation of (a) pore diameter, (b) porosity and (c) pore depth of porous silicon films.

The pore diameter, porosity and pore depth were obtained from the analysis of the top view and cross sectional SEM images, repectively. In the measurements of pore diameter

and pore depth using ImageJ®, a calibration was set by using the scale bar of known length appearing on the right bottom corner of the SEM image. Average pore diameter (d_{avg}) and pore depth (h_{avg}) with standard derivations were computed by analyzing more than 40 different pores (indicated with white lines shown in Fig. S1 a and c on each image and using more than 3 images collected from different regionsbut at same radial distance from the center of the circular region. For porosity (p) calculations, the porous area was selected by adjusting the slider bar on the "Threshold" menu Fig. S1 b. "Analyze Particles" tool was then used to calculate and display the area fraction value, which represents percent porosity. The average porosity was determined by repeating this analysis 3 times for each image and using 3 different images at locations equidistant from the center of the circular region.

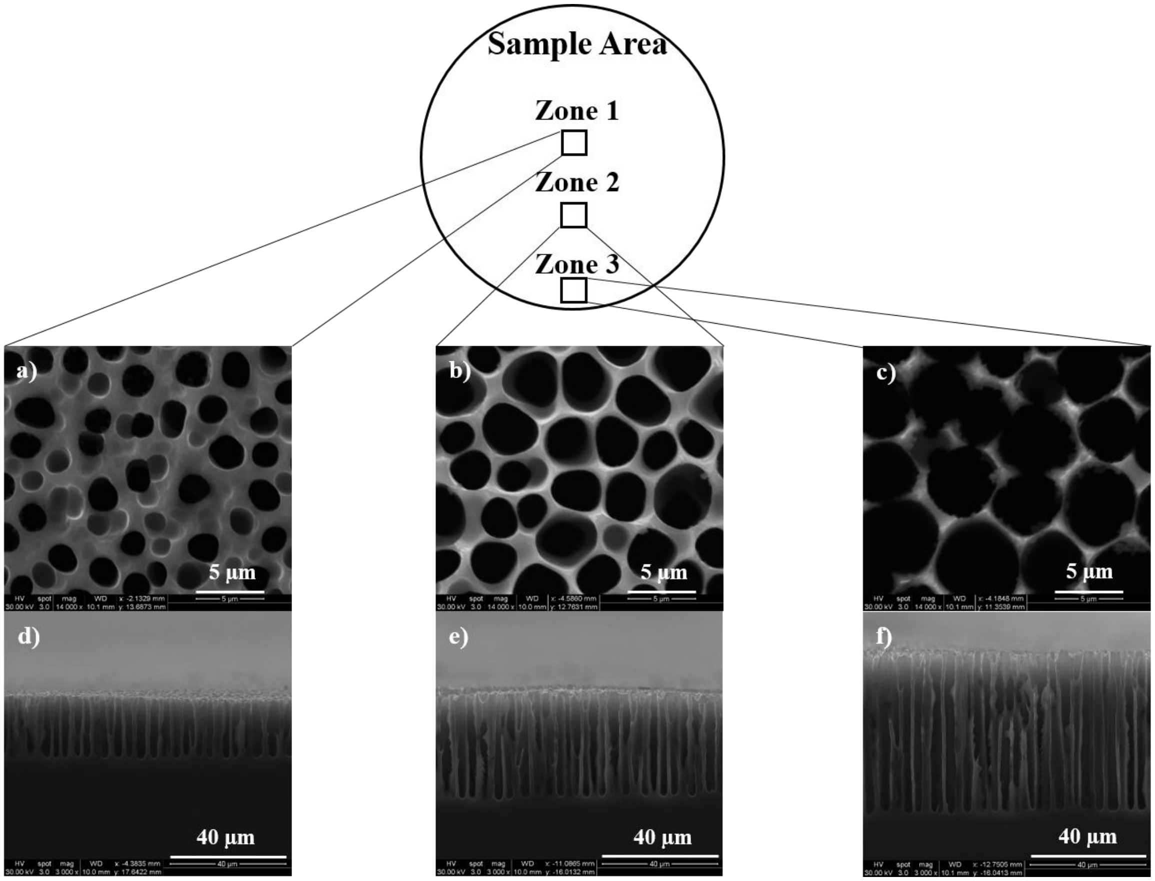

Supplementary Figure 2. Surface morphology of porous silicon samples prepared using etchant solution containing 3E-3% Triton® X-100. SEM images showing (a)-(c) top and (d)-(f) cross sectional view of porous films at Zone 1, 2 and 3.

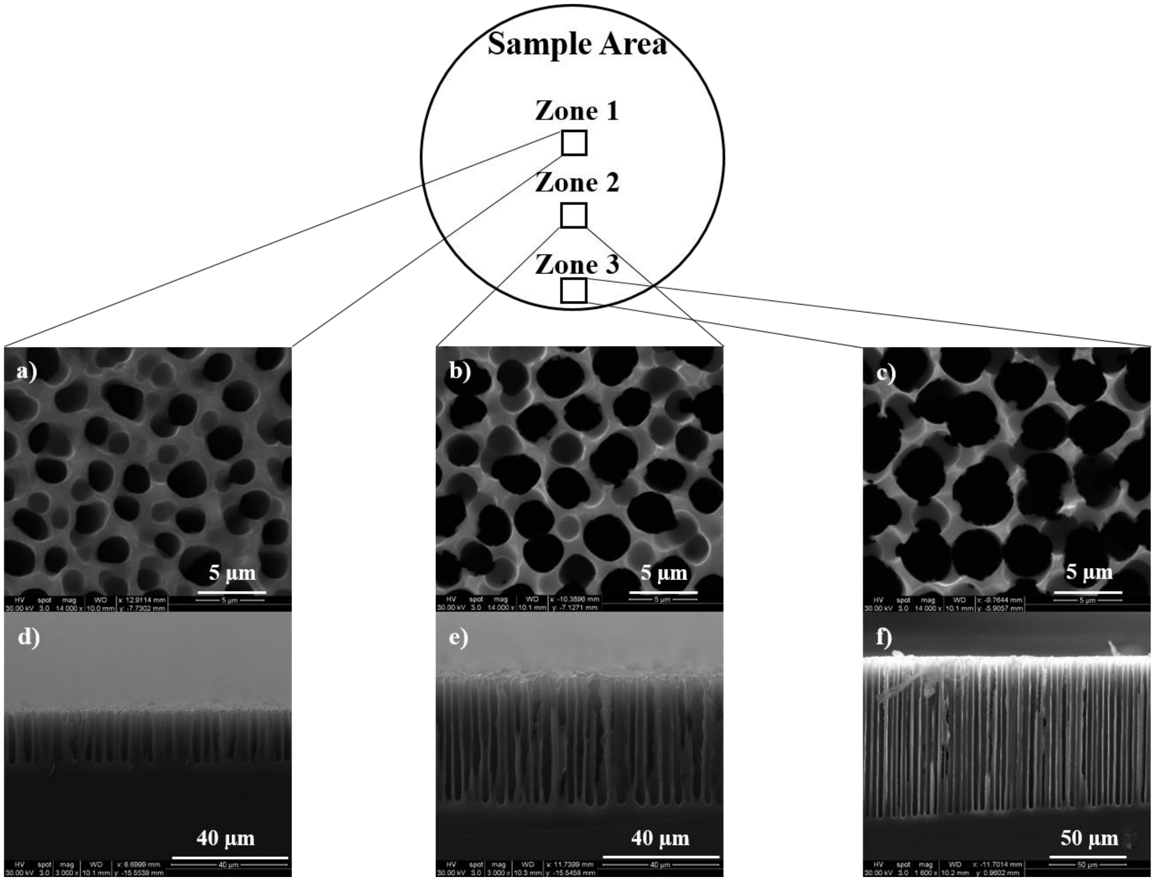

Supplementary Figure 3. Surface morphology of porous silicon samples prepared using etchant solution containing 9E-3% Triton® X-100. SEM images showing (a)-(c) top and (d)-(f) cross sectional view of porous films at Zone 1, 2 and 3.

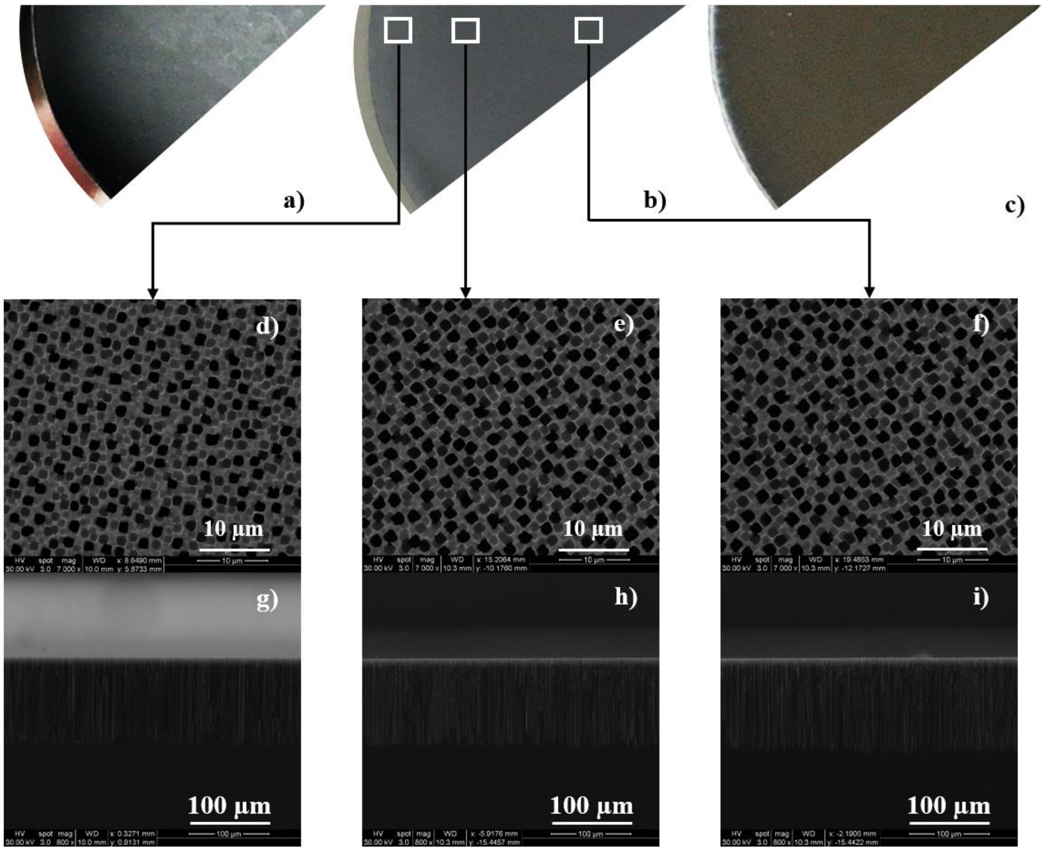

Supplementary Figure 4. Comparison of porous film morphology under different conditions. Photographs showing color gradient on the actual samples prepared using etchant solutions containing (a): 1E-1% Triton® X-100 and (b) and (c): 84% DMSO. (d) – (e) top view and (g) – (f) cross sectional view of SEM images for sample (prepared using 84 % DMSO) showing homogeneous porous films with similar porosity, pore size and pore depth values radially along the sample. Wafer resistivity: (a) and (b): 14 Ω-cm; (c): 8 Ω-cm.

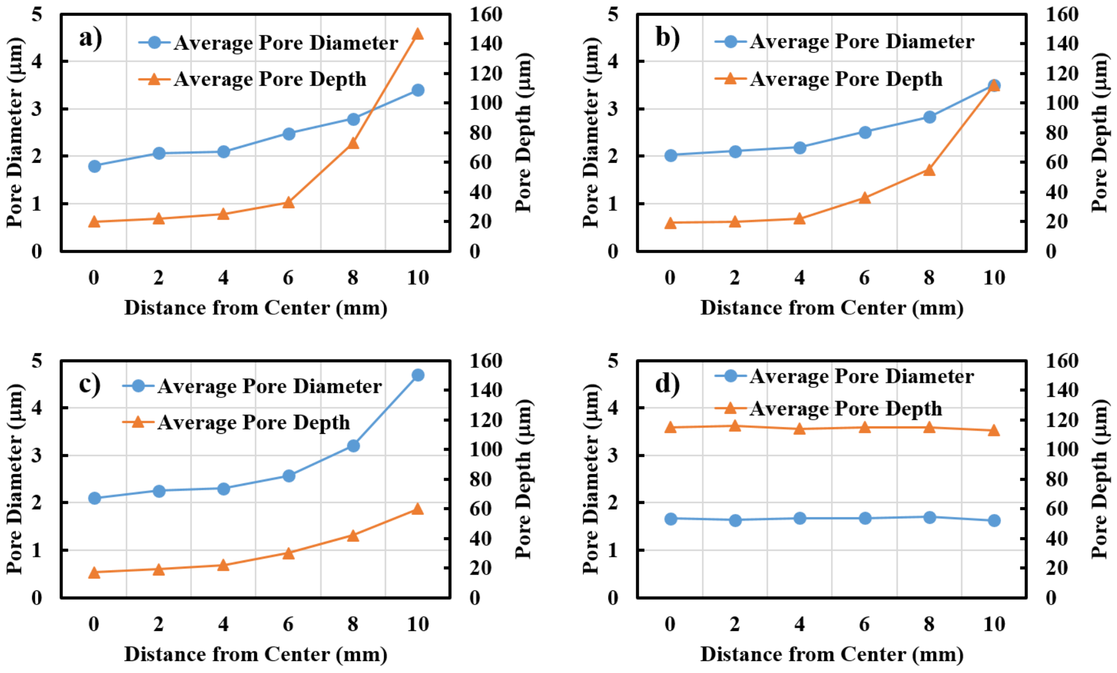

Supplementary Figure 5. Comparison of porous film characteristics under different conditions. Plots showing pore diameter (primary Y axis) and pore depth (secondary Y axis) as a function of distance from the center of the sample prepared using (a) 8% HF/water/1E-1% Triton® X-100, (b) 8% HF/water/9E-3% Triton® X-100, (c) 8% HF/water/3E-3% Triton® X-100 and (d) 8% HF/8% H_2O/84% DMSO as etching solutions.

Supplementary Table 1. Results of pore dimension analysis. Morphological characteristics for porous films obtained using different concentrations of Triton® X-100 in the etchant solution.

Triton® X-100 Concentration	Zone	Average pore size (d_{avg}, μm)	Average pore depth (h_{avg}, μm)	Porosity (p)
**3E-3%**	**1**	2.1±0.3	20±1	21.1%
	**2**	3.3±0.7	32±2	58.5%
	**3**	4.7±0.8	53±2	75.2%
**9E-3%**	**1**	2.1±0.4	19±1	25.6%
	**2**	2.9±0.4	41±4	48.9%
	**3**	3.5±0.4	97±4	61.0%
**1E-1%**	**1**	1.8±0.2	23±1	19.3%
	**2**	3.1±0.4	58±5	52.4%
	**3**	3.4±0.5	127±7	63.2%
